# Situational Analysis of Cancer Care Services in India: Protocol for a Nationwide Cross-Sectional Health Systems Study

**DOI:** 10.2196/99765

**Published:** 2026-10-21

**Authors:** Anita Nath, Abhishek David George, Sudarshan K L, Krishnan Sathishkumar, Thilagavathi Ramamoorthy, Bency Joseph, Gokul Sarveswaran, Prashant Mathur

**Affiliations:** 1 Indian Council of Medical Research-National Institute of NCD Epidemiology, Poojanahalli Road, Kanamangala P.O, Devanahalli Taluk, Bengaluru, Karnataka, 562110, India, 91 9972384877; 2 Impressive Hospitalities Pvt Ltd, Hosted at Indian Council of Medical Research-National Institute of NCD Epidemiology, Bengaluru, India

**Keywords:** oncology service, health care facilities, cancer care facilities, India, health system

## Abstract

**Background:**

Cancer is a significant public health challenge in India, contributing to 8.3% of deaths and 5.0% of disability-adjusted life years in 2016—nearly twice its contribution in 1990. Fragmented service delivery, limited specialist availability, late presentation, and significant financial hardship continue to hinder access to timely and effective cancer care, particularly in rural and underserved regions. A comprehensive, system-level assessment is essential to inform evidence-based planning and strengthen cancer services nationwide.

**Objective:**

This study aims to assess the availability, readiness, accessibility, and quality of cancer care services across primary, secondary, and tertiary health care facilities in India. Specifically, it will evaluate infrastructure, human resources, diagnostic and treatment capacity, referral pathways, and service delivery across the 6 Institute of Medicine quality domains (equitable, effective, patient-centered, safe, efficient, and timely); compare rural-urban and public-private disparities; and use these findings to develop an evidence-informed framework for strengthening cancer care services and informing national policy and program planning.

**Methods:**

A 3-year cross-sectional descriptive study was conducted across 31 states and union territories using proportionate district sampling based on rural-urban population distribution. Nodal hospitals coordinated data collection from primary, secondary, and tertiary health care facilities. Facility-level data were collected using 3 standardized electronic forms—developed separately for primary, secondary, and tertiary levels of care—covering infrastructure, workforce, diagnostic and treatment services, referral systems, and service. Descriptive statistics will be used to summarize service availability and readiness, and chi-square tests to compare rural-urban, public-private, and facility-level differences.

**Results:**

The study received funding in October 2023. Facility-level data collection took place between July 2024 and March 2026. We enrolled 6896 health facilities (4353 primary, 1979 secondary, and 564 tertiary) from 352 districts across 31 states and union territories, with participation rates of 64.6% (4353/6735) for primary health centers, 75.7% (1979/2612) for secondary-level facilities, and 63.9% (564/902) for tertiary hospitals. Data cleaning and quality checks are complete, and analysis is complete but unpublished; we will report results on service availability, readiness, and regional variation separately.

**Conclusions:**

This nationwide assessment is expected to provide one of the most comprehensive evaluations of cancer care preparedness undertaken in India. Findings are anticipated to inform national policy and program planning, including investment priorities under the NP-NCD, Ayushman Bharat, and related initiatives to support progress toward equitable, comprehensive cancer care and universal health coverage.

## Introduction

Cancer accounted for 8.3% of deaths and 5.0% of disability-adjusted life years in India in 2016, twice its contribution in 1990 [1]. Delayed cancer diagnosis and treatment are often responsible for a significant proportion of cancer deaths, as survival depends mainly on the stage of the disease at the time of diagnosis. In many low- and middle-income countries (LMICs), including India, cancer care systems remain fragmented and inadequately structured, largely due to financial constraints, social disparities, and logistical challenges [2,3]. Cancer care in rural India is hindered by low awareness, late presentation, and limited access to diagnostic and specialist services [4]. The costs associated with cancer diagnosis, treatment, and supportive services often impose severe economic strain, resulting in catastrophic health expenditures that can drive households into poverty [5]. The burden of cancer in India is linked to inequities in health care access and uneven distribution of infrastructure and human resources across the country. A report by the World Cancer Initiative on the “Cancer Preparedness Index” ranked India 19th among the 28 countries included in the study [6].

Regarding the health care access and quality index, India ranked 145th out of 195 countries and territories in 2016, compared with neighboring China (48th), Sri Lanka (71st), and Bangladesh (132nd) [7]. As much as half of all premature deaths from noncommunicable diseases (NCDs), including cancer, have been linked to weaknesses in health systems that fail to provide timely, equitable, and effective care to the population [8]. A consensus is that about 60% of cancer deaths can be averted with adequate preventive measures and early detection [9].

Sustainable development goal 3 emphasizes ensuring healthy lives and promoting well-being for all [10]. A key target under this goal is to enhance mental health and well-being and to achieve a one-third reduction in premature mortality from NCDs by 2030 through effective prevention and treatment strategies. The Government of India launched the National Programme for Prevention and Control of Non-Communicable Diseases (NP-NCD) in response to the growing burden of NCDs in the country. As part of the Ayushman Bharat Pradhan Mantri Jan Arogya Yojana, the nation’s commitment to finance cancer care is paving the way for “patient-centered” care. Telemedicine has transformed health care delivery across multiple specialties and holds significant promise for strengthening the continuum of cancer care in India [11].

According to the World Health Organization, cancer control encompasses 4 key components: prevention, early detection, diagnosis and treatment, and palliative care [12]. Various factors can affect the quality of the health care delivery system, including resources, facility capacity, health care provider competence, and the public-private distribution of health services [13]. The revised NP-NCD operational guidelines provide a broader framework to facilitate the effective implementation of the program; however, they include only a few key monitoring indicators and targets relevant to cancer control [14]. Cancer care centers serve as pivotal institutions for delivering comprehensive cancer services and for implementing cancer control programs.

Despite ongoing investments in cancer control, comprehensive evidence on the readiness of India’s health system to deliver equitable cancer care across the continuum of prevention, early detection, diagnosis, treatment, survivorship, and palliative care remains limited. Existing assessments have largely focused on individual services or specific levels of care, with few studies adopting a systems perspective to evaluate preparedness across primary, secondary, and tertiary health care facilities. The assessment was further informed by the Institute of Medicine’s 6 domains of health care quality (safety, effectiveness, patient-centeredness, timeliness, efficiency, and equity) and the Indian Public Health Standards (IPHS) 2022, which define the expected infrastructure, human resources, equipment, and service delivery standards across different levels of care [13-15]. Together, these frameworks provide a comprehensive basis for evaluating cancer care readiness and identifying health system gaps that may influence equitable access to quality cancer services. Therefore, the objectives of this study were (1) to assess the availability and preparedness of cancer prevention, screening, diagnostic, treatment, survivorship, supportive care, and patient safety services across primary, secondary, and tertiary health care facilities in India; (2) to examine regional, rural-urban, and public-private variations in cancer care availability; and (3) to identify critical gaps in infrastructure, human resources, and service delivery to inform evidence-based planning and strengthening of cancer care in India.

## Methods

### Study Design

This study adopted a cross-sectional, descriptive design implemented in 3 phases. It was conducted across 31 states and union territories, using a proportionate sampling approach to select districts from each state and ensure representativeness across all 3 district categories—rural, rural-urban, or urban—based on Census 2011 population distribution, as shown in Figure 1.

**Figure 1.**
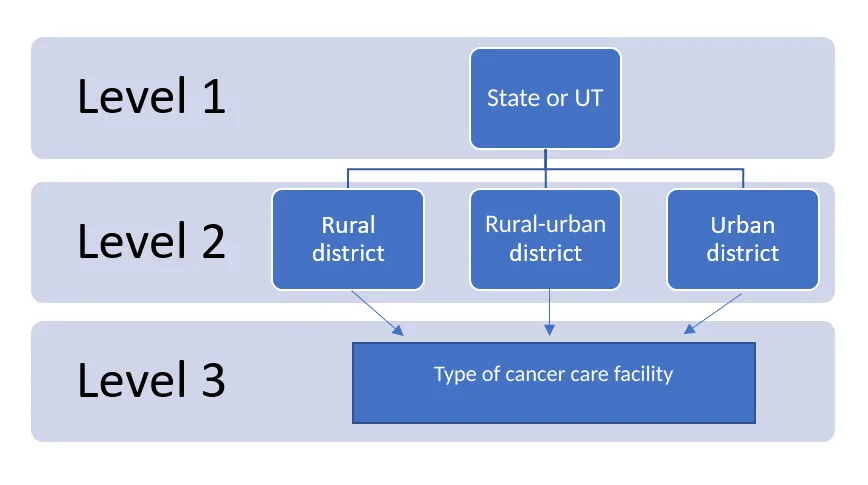
Stratification of study area and sampling frame. UT: union territory.

### Study Setting and Sampling

Nodal hospitals are central hub institutions—often designated by the Ministry of Health and Family Welfare—that coordinate care, surveillance, or service delivery for a specific disease area, public health program, or specialized service within a defined region. Depending on the number of districts in each state and union territory, nodal hospitals were identified [14]. Preference was given to institutions hosting a hospital-based cancer registry under the National Cancer Registry Programme (NCRP), as these centers possess established expertise in cancer registration, surveillance, and clinical oncology. In addition, hospitals recognized as centers of excellence in cancer care, with adequate infrastructure, multidisciplinary oncology services, and experience in coordinating multicentric research activities, were preferentially selected.

These selected nodal hospitals implemented data collection within the region, and each covered a defined number of districts. Table 1 summarizes the sampling framework that underpinned the study’s representativeness. The proportionate district selection strategy was designed to capture the diversity of health care delivery across states and union territories while ensuring adequate representation of rural, urban, and mixed districts [13]. Inclusion of primary, secondary, and tertiary health care facilities within each sampled district enabled assessment of cancer care across the continuum of service delivery and facilitated comparisons of regional, geographic, and health system variations. In smaller states (≤10 districts), we included all districts. In larger states, 8 to 50 districts were selected using proportionate sampling. This sampling approach supported the study objectives of evaluating the availability, preparedness, and geographic distribution of cancer care services across India.

**Table 1.** Sampling framework for district and health facility selection based on state size for cancer care services assessment.

Total number of districts per State	Minimum number of districts to be covered	District stratification	Health facilities
		Rural and urban	Primary^a^, secondary^b^, and tertiary^c^ level
<10	All		
11‐20	8‐10		
21‐30	10‐15		
31-40	15-20		
41-50	20-25		
51-60	25-30		
>60	30-50		

a Primary level: primary health centers.

b Secondary level: community health centers, subdistrict hospitals, and district hospitals.

c Tertiary level: medical colleges, regional cancer centers, specialized cancer institutions (public and private), and multispecialty private hospitals providing cancer care.

### Data Collection Tools and Domains

Data were collected using 3 standardized, facility-specific electronic forms developed for primary (primary health centers), secondary (community health centers, subdistrict hospitals, and district hospitals), and tertiary health care facilities (regional cancer centers, tertiary cancer care centers, medical college hospitals, superspecialty hospitals, and private multispecialty hospitals), as shown in Figure 2. Each questionnaire comprised structured questions with predefined response options tailored to the services and infrastructure relevant to that level of care. The study investigators developed the forms through expert consultation and aligned them with the IPHS 2022 for the respective facility levels and the Institute of Medicine’s 6 domains of health care quality. The complete data collection instruments are provided as Multimedia Appendix 1.

**Figure 2.**
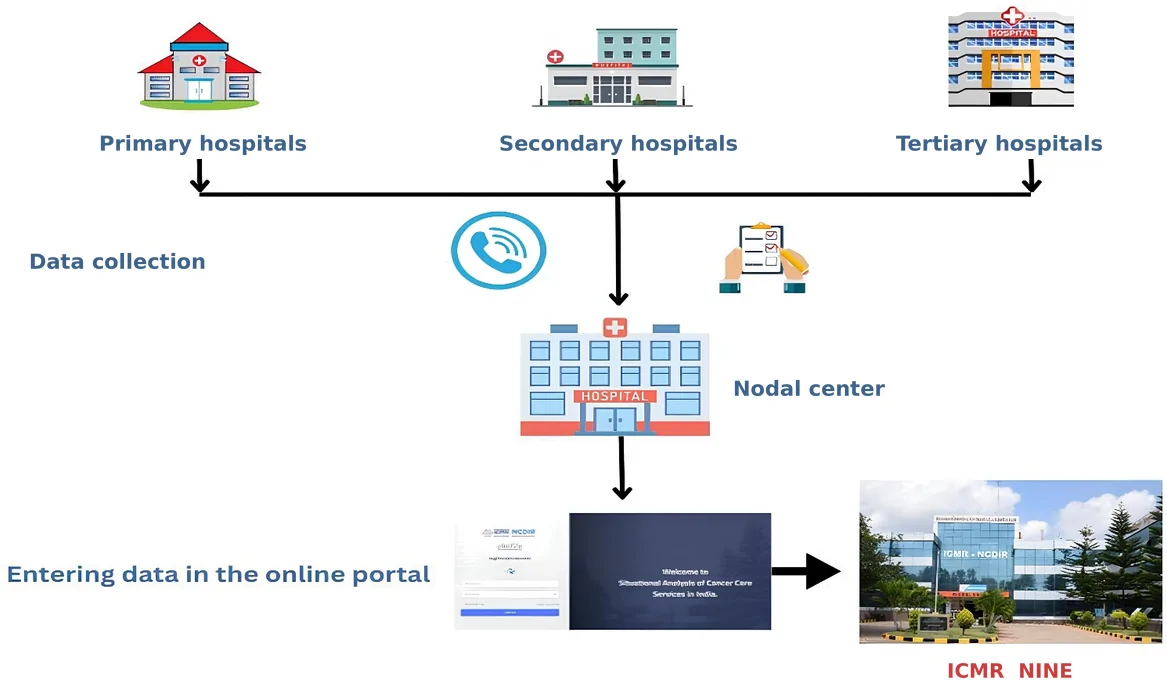
Flow of data collection and transmission. ICMR NCDIR: Indian Council of Medical Research National Institute of NCD Epidemiology

We developed a secure web-based data collection portal for the study. Site investigators at each nodal institution received unique login credentials to access the portal, which included a list of eligible health care facilities within their assigned districts. Facility-specific electronic questionnaires were made available according to the level of care. Data were collected from designated key informants, including medical superintendents, hospital administrators, heads of departments, or senior clinicians familiar with cancer care services at the facility. Site investigators coordinated with the identified facilities, obtained the required information from the designated respondents, and entered the responses into the online portal displayed in Figure 3. Built-in validation rules, mandatory fields, skip logic, and range checks were incorporated to minimize data entry errors and ensure the completeness and consistency of the submitted data.

**Figure 3.**
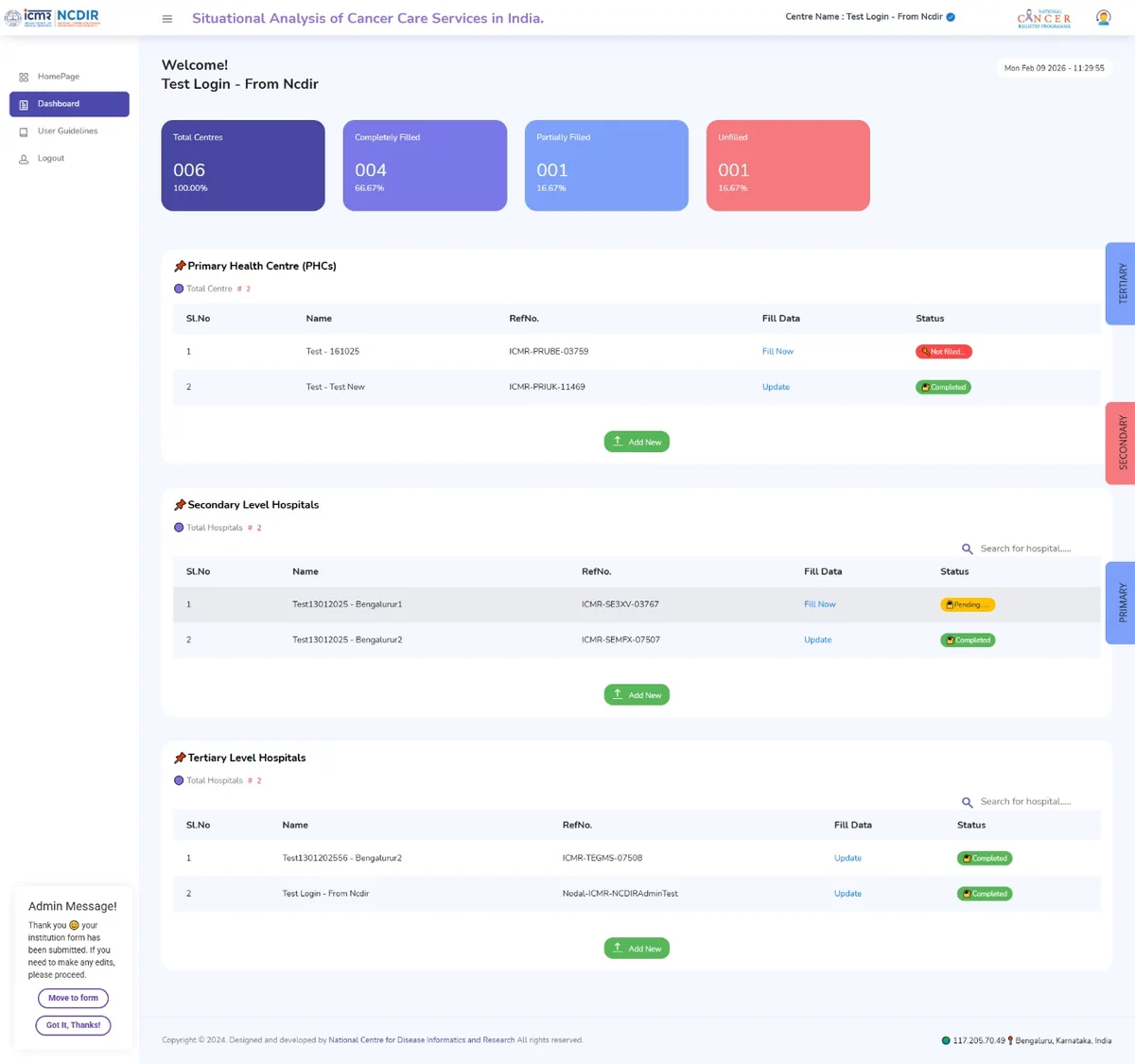
Electronic data capture portal.

The assessment captured information across the 6 domains of health care quality—equity, effectiveness, patient-centeredness, safety, efficiency, and timeliness—based on the Institute of Medicine framework. To ensure that the assessment reflected the expected standards of service delivery at each level of the health system, the indicators were also mapped to the IPHS 2022, which specify the essential and desirable requirements for infrastructure, human resources, equipment, diagnostics, and service delivery across primary, secondary, and tertiary health care facilities in India.

All 3 forms collected information on general facility characteristics (facility type, ownership, location, bed strength where applicable, annual patient volume, and respondent details) and the availability of cancer prevention, screening, diagnostic, treatment, human resource, patient-centered care, and safety services, although the scope of information varied according to the level of care.

The primary health care facility questionnaire assessed the availability of preventive and early detection services (human papillomavirus vaccination, tobacco cessation, and cervical and breast cancer screening), basic laboratory diagnostic services, referral mechanisms, and essential infrastructure and workforce required for cancer prevention and early detection. The secondary health care facility questionnaire additionally captured information on pathology and imaging services, cancer treatment (including surgery and chemotherapy where available), availability of cancer-related health care professionals, patient-centered care practices, and institutional safety measures such as chemotherapy handling protocols, monitoring of adverse events, and infection prevention practices. The tertiary health care facility questionnaire included a comprehensive assessment of advanced diagnostic services, multimodality cancer treatment (surgery, systemic therapy, radiotherapy, and palliative and survivorship care), supportive care services, multidisciplinary team functioning, adherence to clinical practice guidelines, specialist oncology workforce, patient safety systems, health information systems, cancer registries, telemedicine services, and digital health infrastructure. Table 2 presents the conceptual framework linking the study objectives to the corresponding outcomes, indicators, data sources, and measurement methods. This framework ensured that data collection remained aligned with the study objectives and enabled systematic assessment of cancer care preparedness across infrastructure, human resources, service delivery, and quality-of-care domains at different levels of the health system.

**Table 2.** Study objectives, outcomes, indicators, data sources, and methods of measurement.

Study objective	Outcome	Indicators	Data source	Method of measurement
Assess availability and preparedness of cancer prevention, screening, diagnostic, treatment, survivorship, supportive care, and patient safety services across primary, secondary, and tertiary health care facilities	Availability and preparedness of cancer care services	Facility infrastructure, prevention and screening, diagnostics, treatment, survivorship, supportive care, patient safety, and health information systems	Facility-specific electronic forms completed by designated key informants	Availability recorded as yes or no or categorical responses and summarized as frequencies and percentages by level of care
Examine regional, rural-urban, and public-private variations in cancer care availability	Variation in cancer care availability	Region, rural or urban location, public or private ownership, facility level, and service availability across all domains	Facility-specific electronic forms	Comparative analyses of proportions across regions, sectors, and facility levels
Identify critical gaps in infrastructure, human resources, and service delivery to inform evidence-based planning and strengthening of cancer care in India	Cancer care readiness and health system gaps	Infrastructure, equipment, oncology workforce, referral pathways, multidisciplinary care, and quality domains	Facility-specific electronic forms aligned with Indian Public Health Standards 2022 and Institute of Medicine quality domains	Gap analysis using domain-specific indicators

### Data Management

Each site had a designated site principal investigator responsible for coordinating data collection. Data were collected from health facilities by identifying a key person at each facility. Data collection was conducted by sharing the study proforma in editable Microsoft Word format or by conducting direct telephone surveys with the designated key personnel. Once the data were collected and verified, they were entered into the secure online portal managed by ICMR-NINE (Indian Council of Medical Research National Institute of NCD Epidemiology, previously known as the National Centre for Disease Informatics and Research). Data entry was centrally monitored through real-time dashboards to ensure completeness and accuracy.

### Data Analysis

Facility-level data collection took place between July 2024 and March 2026. We analyzed data according to the predefined study objectives and outcome indicators presented in Table 2. Descriptive statistics, including frequencies and percentages, summarized the availability of infrastructure, human resources, cancer prevention, screening, diagnostic, treatment, survivorship, supportive care, patient safety, and health information system components across primary, secondary, and tertiary health care facilities. We summarized service availability by geographic region, level of health care, rural-urban location, and ownership (public or private), enabling direct comparison of health system preparedness across settings.

Comparative analyses examined regional, rural-urban, and public-private differences in cancer care service availability. We used the Pearson chi-square test to assess statistically significant differences in categorical variables, with a 2-sided *P* value <.05 considered statistically significant. Data cleaning, quality checks, and analysis are complete but unpublished; we will report results on service availability, readiness, and regional variation separately.

### Ethical Considerations

The study protocol was submitted to and reviewed by the institutional ethics committee (IEC) of the National Centre for Disease Informatics and Research, now the ICMR-NINE, which coordinated the study. The IEC determined that the protocol was exempt from full ethics review in accordance with the ICMR National Ethical Guidelines for Biomedical and Health Research Involving Human Participants (2017) [16], as it involved a facility-level assessment of health system preparedness conducted as part of a public health program evaluation, posed less than minimal risk to participants, and did not involve collection of patient-level identifiable information (NCDIR/IEC/3058/2022).

## Results

The study received funding in October 2023. Facility-level data collection took place between July 2024 and March 2026. A total of 4353 primary health centers (PHCs), 1979 secondary-level health facilities, and 564 tertiary hospitals from 352 districts across 31 states and union territories participated in the assessment (Table 3). Overall participation rates were 64.6% (4353/6735) for PHCs, 75.7% (1979/2612) for secondary-level facilities, and 63.9% (564/902) for tertiary hospitals. Most participating PHCs were located in rural areas (3786/4353, 87%). District coverage was highest in the east (76/77, 98.7%) and central (105/108, 97.2%) regions, while coverage was lower in the north (37/72, 51.4%) and south (32/79, 40.5%) regions. The broad geographic coverage achieved across 352 districts and all 3 levels of health care provided a comprehensive overview of the current organization and preparedness of cancer care services in India. Including facilities from diverse geographic regions, rural and urban settings, and both public and private sectors ensured that the assessment adequately addressed the study objective of evaluating variations in service availability across the health system.

Data cleaning and quality checks are complete, and analysis is complete but unpublished; results on service availability, readiness, and regional variation will be reported separately.

**Table 3.** Study status and sample achieved.

Region	Districts	Primary health centers			Secondary health facilities			Tertiary health facilities				
	Covered, 352/451(%)	Covered, 4353/6735 (%)	Rural, 3786/4353 (%)	Urban, 567/4353 (%)	Covered, 1979/2612 (%)	Rural, 1111/1979 (%)	Urban, 868/1979 (%)	Covered, 564/902 (%)	Rural, 56/564 (%)	Urban, 508/564 (%)	Public, 204/564 (%)	Private, 360/564 (%)
North	37/72 (51.4)	491/1125 (43.6)	395/491 (80.4)	96/491 (19.6)	295/375 (78.7)	169/295 (57.3)	126/295 (42.7)	110/144 (76.4)	7/110 (6.4)	103/110 (93.6)	35/110 (31.8)	75/110 (68.2)
East	76/77 (98.7)	801/1155 (69.4)	712/801 (88.9)	89/801 (11.1)	435/539 (80.7)	248/435 (57)	187/435 (43)	95/154 (61.7)	7/95 (7.4)	88/95 (92.6)	42/95 (44.2)	53/95 (55.8)
South	32/79 (40.5)	370/1110 (33.3)	301/370 (81.4)	69/370 (18.6)	166/370 (44.9)	97/166 (58.4)	69/166 (41.6)	99/158 (62.7)	6/99 (6.1)	93/99 (93.9)	27/99 (27.3)	72/99 (72.7)
West	36/40 (90)	458/600 (76.3)	434/458 (94.8)	24/458 (5.2)	133/200 (66.5)	73/133 (54.9)	60/133 (45.1)	44/80 (55)	5/44 (11.4)	39/44 (88.6)	24/44 (54.5)	20/44 (45.5)
Central	105/108 (97.2)	1407/1620 (86.9)	1206/1407 (85.7)	201/1407 (14.3)	647/756 (85.6)	332/647 (51.3)	315/647 (48.7)	143/216 (66.2)	19/143 (13.3)	124/143 (86.7)	49/143 (34.3)	94/143 (65.7)
Northeast	66/75 (88)	826/1125 (73.4)	738/826 (89.3)	88/826 (10.7)	303/372 (81.5)	192/303 (63.4)	111/303 (36.6)	73/150 (48.7)	12/73 (16.4)	61/73 (83.6)	27/73 (37)	46/73 (63)

## Discussion

This study is one of the largest nationwide assessments of cancer care preparedness in India across primary, secondary, and tertiary health care facilities, using a standardized health systems framework. Preliminary analyses demonstrate broad participation from health care facilities across diverse geographical regions and highlight substantial regional, rural-urban, and public-private variation in the availability of cancer prevention, screening, diagnostic, treatment, survivorship, and patient safety services. Existing evidence on cancer care capacity in India largely comes from situational assessments focused on individual states or specific components of care. For example, Raj et al [17] reported important deficiencies in oncology infrastructure and workforce in Bihar, highlighting shortages in specialized human resources and treatment facilities. Similarly, a national survey of National Cancer Grid centers identified substantial variation in palliative care services despite these being specialized oncology institutions, with deficiencies in trained personnel, supportive care services, funding, and institutional quality systems [18]. Nationwide assessments encompassing the entire continuum of cancer care across all levels of the health system have, however, remained limited. By including primary, secondary, and tertiary health care facilities from most states and union territories, this study aims to extend existing evidence and provide a more comprehensive evaluation of health system preparedness than previously available.

The study is also expected to complement findings from the NCRP, which has documented considerable differences in cancer incidence, mortality, and survival across India, reflecting variation in health care infrastructure, specialist workforce, and access to timely diagnosis and treatment [19,20]. Projections indicate that India’s growing cancer burden will continue to increase demand for comprehensive cancer services, reinforcing the need for system-level evidence to guide capacity strengthening [21]. Similar challenges have been reported globally, with studies from LMICs highlighting inequitable access to diagnostics, specialist care, multidisciplinary treatment, and referral systems as major determinants of cancer outcomes [22,23]. Findings from this study are therefore anticipated to contribute to a broader understanding of how health system preparedness shapes cancer outcomes, both within India and in comparable settings.

The findings are expected to have direct implications for cancer control planning in India, encompassing prevention, early detection, diagnosis, treatment, survivorship, and palliative care services, rather than treatment alone. Should regional disparities in service availability emerge, as suggested by prior state- and institution-level assessments, this would indicate that investment needs to extend beyond tertiary cancer centers to strengthen primary and secondary health care services, particularly for prevention, screening, pathology, imaging, referral mechanisms, and specialist workforce development. Assessing preparedness across the 6 recognized domains of health care quality will enable evaluation beyond infrastructure alone, while including both public and private facilities and rural and urban settings will help identify geographic and sectoral inequities relevant to policy planning. The study is designed to complement ongoing national initiatives under the NP-NCD, Ayushman Bharat, and the recently approved Day Care Cancer Centers. Martin-Moreno et al [24] have emphasized that integrated and timely cancer care improves survival while reducing the financial and social burden on patients and their families; the evidence generated through this study is intended to support policymakers in prioritizing investments according to regional needs and strengthening equitable access to comprehensive cancer care.

A major strength of this study is its planned nationwide coverage, encompassing more than 6800 health care facilities across 352 districts and representing all major geographical regions and all 3 levels of health care. The use of standardized facility-specific assessment tools aligned with the IPHS 2022 and internationally recognized health care quality domains is intended to enable systematic comparison across diverse health care settings. Inclusion of both public and private facilities is expected to enhance the comprehensiveness of the assessment and provide a robust baseline for future monitoring of cancer care preparedness.

Certain limitations should also be acknowledged. First, the assessment was cross-sectional and therefore reflected service availability at a single point in time. Second, information was obtained from designated facility respondents and was not independently verified through direct facility observation, introducing the possibility of reporting bias. Third, although the sampling strategy aimed to achieve broad national representation, districts varied across regions, and participation may have been lower in some states because of operational constraints. Finally, the study assessed service availability and preparedness, not service use, quality of clinical care, patient outcomes, or treatment effectiveness.

Planned future work includes developing composite preparedness indicators, evaluating regional disparities in detail, and exploring determinants associated with variation in cancer care capacity. Linkage of study findings with cancer registry data, treatment outcomes, and health financing information may provide further insight into the relationship between health system preparedness and cancer outcomes. Repeated assessments using the same framework could also help monitor progress after implementing national cancer control initiatives and future investments in cancer care infrastructure.

## Supplementary material

10.2196/99765 Multimedia Appendix 1 Study proforma.

10.2196/99765 Checklist 1 STROBE checklist.

## Abbreviations

ICMR-NINE: Indian Council of Medical Research National Institute of NCD Epidemiology
IEC: institutional ethics committee
IPHS: Indian Public Health Standards
LMIC: low- and middle-income country
NCD: noncommunicable disease
NCRP: National Cancer Registry Programme
NP-NCD: National Programme for Prevention and Control of Non-Communicable Diseases
STROBE: Strengthening the Reporting of Observational Studies in Epidemiology
