# Supplementary material for: Situational Analysis of Cancer Care Services in India: Protocol for a Nationwide Cross-Sectional Health Systems Study
Source: JMIR Res Protoc. 2026 Oct 21;15:e99765. doi: 10.2196/99765 (PMC13686612; doi:10.2196/99765)
Supplement: Supplementary file 1 [file resprot-v15-e99765-s001.docx]

Supplementary material

MULTIMEDIA Appendix 1

*STUDY PROFORMA**

| **Respondent details** | | Name |  | | |  |  |  |  |  |  |  |
| --- | --- | --- | --- | --- | --- | --- | --- | --- | --- | --- | --- | --- |
|  | | Designation |  | | |  |  |  |  |  |  |  |
|  | | Contact Phone number |  | | |  |  |  |  |  |  |  |
|  | | Email id |  | | |  |  |  |  |  |  |  |
| **Date of recording the information** | |  |  | | |  |  |  |  |  |  |  |
| A. **GENERAL INFORMATION** | | | | | | | | | | | | |
| 1 | Name of the Health Facility | | |  | | | | | | | | |
| 2. | Type of Health Facility  (Select any one option) | | | Super speciality | | | If yes | | Government | | |  |
|  |  |  |  |  |  |  |  |  | Private (including NGO managed/charitable) | | |  |
|  |  |  |  |  |  |  |  |  | Mixed | | |  |
|  |  |  |  | Medical College | | | If yes | | Government | | |  |
|  |  |  |  |  |  |  |  |  | Private (including NGO managed/charitable) | | |  |
|  |  |  |  |  |  |  |  |  | Mixed | | |  |
|  |  |  |  | Private multi-speciality hospital | | |  | | | | | |
|  |  |  |  | District Hospital | | |  |  |  |  |  |  |
|  |  |  |  | Sub district hospital | | |  |  |  |  |  |  |
|  |  |  |  | Community Health Centre | | |  |  |  |  |  |  |
|  |  |  |  | Primary Health Centre | | |  |  |  |  |  |  |
| 3 | Address of the health facility | | | Urban area (Town/city) | | | Road / Street Name | |  | | | |
|  |  |  |  |  |  |  | Area / Locality | |  | | | |
|  |  |  |  |  |  |  | Ward / Corporation / Division | |  | | | |
|  |  |  |  |  |  |  | Name of City / Town | |  | | | |
|  |  |  |  | Rural area | | | Name of Gram Panchayat/Village | |  | | | |
|  |  |  |  |  |  |  | Name of Sub-Unit of District (Taluk / Tehsil / Other): | |  | | | |
|  |  |  |  | Urban or rural area | | | Name of District | |  | | | |
|  |  |  |  |  |  |  | Postal Pin Code | |  | | | |
| 4 | Total number of facility beds | | | >1000 | | | | | | | | |
|  |  |  |  | 500-1000 | | | | | | | | |
|  |  |  |  | 100-499 | | | | | | | | |
|  |  |  |  | 50-99 | | | | | | | | |
|  |  |  |  | <50 | | | | | | | | |
| 5 | Total number of new cancer patients reporting to the health facility per annum | | |  | | | | | | | | |
| 6 | Total number of unique daycare treatments in the health facility per annum  (If applicable) | | |  | | | | | | | | |
| 7 | Total number of outpatient visits of all cancer patients in the health facility | | |  | | | | | | | | |
| 8 | Super speciality/Medical College/Private multi-speciality hospital:  Under which of the following systems of medicine is cancer care available at this facility? | | | Only Allopathy | | | | | | | | |
|  |  |  |  | Only AYUSH | | | | Ayurveda | | | | |
|  |  |  |  |  |  |  |  | Unani | | | | |
|  |  |  |  |  |  |  |  | Siddha | | | | |
|  |  |  |  |  |  |  |  | Yoga and naturopathy | | | | |
|  |  |  |  |  |  |  |  | Homeopathy | | | | |
|  |  |  |  | Combination of Allopathy and AYUSH | | | | | | | | |
| **B.SERVICE AVAILABILITY** | | | | | | | | | | | |  |
| **B1 CANCER PREVENTION AND SCREENING** | | | | | | | | | | | |  |
| Does this Health facility have the following prevention and screening services? | | | | For : Super speciality/Medical College/Private multi-speciality hospital  (Select: YES/NO)  If YES (In-sourced or Outsourced) | | | | | HPV vaccination | | | |
|  |  |  |  |  |  |  |  |  | Tobacco cessation clinic | | OPD | |
|  |  |  |  |  |  |  |  |  |  |  | Community based | |
|  |  |  |  |  |  |  |  |  | Breast cancer screening | Digital Breast Tomosynthesis | | |
|  |  |  |  |  |  |  |  |  |  | Two-dimensional (2D) mammography | | |
|  |  |  |  |  |  |  |  |  |  | Breast ultra-sound | | |
|  |  |  |  |  |  |  |  |  | Cervical cancer screening | Cervical cytology | | |
|  |  |  |  |  |  |  |  |  |  | VIA | | |
|  |  |  |  |  |  |  |  |  |  | HPV Test | | |
|  |  |  |  |  |  |  |  |  | Oral Cancer screening | | | |
|  |  |  |  | For: District Hospital/Sub district hospital/ Community Health Centre/Primary Health Centre  (Select: YES/NO)  If YES (In-sourced or Outsourced) | | | | | HPV vaccination | | | |
|  |  |  |  |  |  |  |  |  | Tobacco cessation services | | | |
|  |  |  |  |  |  |  |  |  | Cervical cancer screening | | | Cervical cytology |
|  |  |  |  |  |  |  |  |  |  |  |  | VIA |
|  |  |  |  |  |  |  |  |  | Breast cancer screening | | | Breast ultrasound |
|  |  |  |  |  |  |  |  |  |  |  |  | Clinical Breast Examination |
| **B 2 CANCER DIAGNOSIS** | | | | | | | | | | | |  |
| Does this Health facility have the following diagnostic services? | | | | Clinical Laboratory | For : Super speciality/Medical College/Private multi-speciality hospital  (Select: YES/NO)  If YES (In-sourced or Outsourced) | | | | Tumour lysis syndrome panel | | | |
|  |  |  |  |  |  |  |  |  | DIC panel | | | |
|  |  |  |  |  |  |  |  |  | Serum protein electrophoresis | | | |
|  |  |  |  |  |  |  |  |  | Immunoassay test | | | |
|  |  |  |  |  | For: District Hospital/Sub district hospital/ Community Health Centre/Primary Health Centre  (Select: YES/NO)  If YES (In-sourced or Outsourced) | | | | Complete Blood Count | | | |
|  |  |  |  |  |  |  |  |  | Liver Function Test | | | |
|  |  |  |  |  |  |  |  |  | Renal Function Test | | | |
|  |  |  |  |  |  |  |  |  | Alkaline Phosphatase | | | |
|  |  |  |  |  |  |  |  |  | DIC coagulation panel | | | |
|  |  |  |  |  |  |  |  |  | Serum Calcium | | | |
|  |  |  |  | Pathology | For : Super speciality/Medical College/Private multi-speciality hospital  (Select: YES/NO)  If YES (In-sourced or Outsourced) | | | | Histopathology | | | |
|  |  |  |  |  |  |  |  |  | Cytopathology | | | |
|  |  |  |  |  |  |  |  |  | Immunohistochemistry | | | |
|  |  |  |  |  |  |  |  |  | Histochemical stains | | | |
|  |  |  |  |  | For: District Hospital/Sub district hospital/ Community Health Centre  (Select: YES/NO)  If YES (In-source or Outsource) | | | | Histopathology | | | |
|  |  |  |  |  |  |  |  |  | Cytopathology | | | |
|  |  |  |  | Medical imaging | Super speciality/Medical College/Private multi-speciality hospital  (Select: YES/NO)  If YES (In-source or Outsource) | | | | MRI Scan | | | -Average number performed every year |
|  |  |  |  |  |  |  |  |  | CT Scan | | |  |
|  |  |  |  |  |  |  |  |  | Fluoroscopic scanning for image-guided procedure | | |  |
|  |  |  |  |  |  |  |  |  | Digital X-ray | | |  |
|  |  |  |  |  |  |  |  |  | Ultrasound scan | | |  |
|  |  |  |  |  |  |  |  |  | SPECT scan | | |  |
|  |  |  |  |  |  |  |  |  | SPECT-CT scans | | |  |
|  |  |  |  |  |  |  |  |  | PET Scan | | |  |
|  |  |  |  |  |  |  |  |  | PET-CT Scan | | |  |
|  |  |  |  |  | District Hospital  (Select: YES/NO)  If YES (In-source or Outsource) | | | | Ultrasound scan | | |  |
|  |  |  |  |  |  |  |  |  | CT Scan | | |  |
|  |  |  |  |  |  |  |  |  | MRI Scan | | |  |
|  |  |  |  |  |  |  |  |  | Digital X-ray | | |  |
|  |  |  |  |  | Sub-District Hospital/CHC  (Select: YES/NO)  If YES (In-source or Outsource) | | | | Ultrasound scan | | |  |
|  |  | | |  |  | | | | Digital X-ray | | |  |
| **B3 CANCER TREATMENT** | | | | | | | | | | | |  |
| 1 | Does this Health facility have the following treatment services? | | | For : Super speciality/Medical College/Private multi-speciality hospital  (Select: YES/NO) | Cancer surgery | | | | | | | |
|  |  |  |  |  | Systemic therapy | | | | Chemotherapy | | | |
|  |  |  |  |  |  |  |  |  | Hormone therapy | | | |
|  |  |  |  |  |  |  |  |  | Targeted therapy | | | |
|  |  |  |  |  |  |  |  |  | Immunomodulators | | | |
|  |  |  |  |  | Bone Marrow Transplantation | | | | | | | |
|  |  |  |  |  | Radiotherapy | | | | Conventional radiotherapy | | | |
|  |  |  |  |  |  |  |  |  | 3D conformal radiation therapy (3D-CRT) | | | |
|  |  |  |  |  |  |  |  |  | Brachytherapy | | | |
|  |  |  |  |  |  |  |  |  | Intensity modulated radiotherapy | | | |
|  |  |  |  |  |  |  |  |  | Stereotactic radiotherapy | | | |
|  |  |  |  |  | Palliative care | | | | Out-patient clinic | | | |
|  |  |  |  |  |  |  |  |  | In-patient | | | |
|  |  |  |  |  |  |  |  |  | Home care service | | | |
|  |  |  |  |  |  |  |  |  | Community based service | | | |
|  |  |  |  |  | Survivorship care | | | | Patient empowerment and education | | | |
|  |  |  |  |  |  |  |  |  | Management based on symptoms, signs of late effects | | | |
|  |  |  |  |  |  |  |  |  | Fertility services | | | |
|  |  |  |  |  |  |  |  |  | Long-term follow-up monitoring and care | | | |
|  |  |  |  |  |  |  |  |  | Any other | | | |
|  |  |  |  | District Hospital  (Select: YES/NO) | Chemotherapy | | | | | | | |
| 3 | Does this Health facility have the following supportive care services? | | | For : Super speciality/Medical College/Private multi-speciality hospital  (Select: YES/NO) | Psychosocial services  (If YES) | | | | Patient and family education and counselling | | | |
|  |  |  |  |  |  |  |  |  | Support, including for financial burdens, housing, transportation and meal support | | | |
|  |  |  |  |  |  |  |  |  | Support preparing for procedures; assessment and support for treatment adherence | | | |
|  |  |  |  |  | Medical  nutrition therapy (MNT) | | | | | | | |
|  |  |  |  |  | Rehabilitation | | | | Physical therapy | | | |
|  |  |  |  |  |  |  |  |  | Occupational Therapy | | | |
|  |  |  |  |  | Stoma care | | | | | | | |
|  |  |  |  |  | Prosthetic care | | | | | | | |
| 2 | For : Super speciality/Medical College/Private multi-speciality hospital  Does this Health facility adopt a multidisciplinary care approach?  (Select: YES/NO) | | | If Yes, how?  (Select suitable options) | Multidisciplinary clinic | | | | | | | |
|  |  |  |  |  | Multidisciplinary team (MDT) meetings | | | | | | | |
| 3 | For : Super speciality/Medical College/Private multi-speciality hospital  Are any Clinical Practice Guidelines followed for cancer care? | | | If Yes, please select suitable options that are most frequently used  (Select suitable options) | Indian Council of Medical Research (ICMR) | | | | | | | |
|  |  |  |  |  | National Cancer Grid (NCG) | | | | | | | |
|  |  |  |  |  | European Society for Medical Oncology (ESMO) | | | | | | | |
|  |  |  |  |  | National Comprehensive Cancer Network (NCCN) | | | | | | | |
|  |  |  |  |  | National Institute for Health and Care Excellence (NICE) | | | | | | | |
|  |  |  |  |  | American Society for Clinical Oncology (ASCO) | | | | | | | |
|  |  |  |  |  | Any other | | | | | | | |
| **HUMAN RESOURCES** | | | | | | | | | | | | |
| Does the health facility have the following cancer-related health care providers in position at this facility? | | | | For : Super speciality/Medical College/Private multi-speciality hospital  (Select: YES/NO) | Medical oncologist | | | | | | | |
|  |  |  |  |  | Haematologist Oncologist | | | | | | | |
|  |  |  |  |  | Surgical oncologist | | | | | | | |
|  |  |  |  |  | Radiation oncologist | | | | | | | |
|  |  |  |  |  | Palliative care physician | | | | | | | |
|  |  |  |  |  | Paediatric oncologist | | | | | | | |
|  |  |  |  |  | Nurses trained in oncology | | | | | | | |
|  |  |  |  | District Hospital  (Select: YES/NO) | Oncologist | | | | | | | |
|  |  |  |  |  | General physicians with some training in oncology care | | | | | | | |
|  |  |  |  |  | Pathologist | | | | | | | |
| **SAFETY** | | | | | | | | | | | | |
| For : Super speciality/Medical College/Private multi-speciality hospital/District Hospital  Does this Health facility have the following risk management plans?  (Select: YES/NO) | | | | Safety protocols for handling chemotherapy drugs | | | | | | | | |
|  |  |  |  | Systemic monitoring and analysis of major adverse or undesirable events | | | | | | | | |
|  |  |  |  | Control of hospital acquired infection | | | | | | | | |
|  |  |  |  | Any other | | | | | | | | |
| **PATIENT CENTERED CARE** | | | | | | | | | | | |  |
| For : Super speciality/Medical College/Private multi-speciality hospital/District Hospital | | | | Is there a staff member appointed as a contact person or “case manager” for each patient?  (Select: YES/NO) | | | | | | | | |
|  |  |  |  | Do patients or caregivers participate in the diagnostic and treatment process for example by having insight in the treatment Timely plan and health data?  (Select: YES/NO) | | | | | | | | |
|  |  |  |  | Are patient reminders sent regarding an coming up hospital visit?  (Select: YES/NO) | If Yes, How | | | | Email | | | |
|  |  |  |  |  |  |  |  |  | Text Message | | | |
|  |  |  |  |  |  |  |  |  | Phone | | | |
| **TIMELINESS** | | | | | | | | | | | |  |
| For : Super speciality/Medical College/Private multi-speciality hospital/District Hospital | | | | Average waiting time between making an appointment and first visit to the health facility | | | | | Within one day | | | |
|  |  |  |  |  |  |  |  |  | 1-3 days | | | |
|  |  |  |  |  |  |  |  |  | 4-7 days | | | |
|  |  |  |  |  |  |  |  |  | >7 days | | | |
|  |  |  |  | Average waiting time between first visit and diagnosis | | | | | Within 1 week | | | |
|  |  |  |  |  |  |  |  |  | 1-2 weeks | | | |
|  |  |  |  |  |  |  |  |  | >2 weeks | | | |
|  |  |  |  | Average waiting time between establishing diagnosis and first treatment | | | | | Within 1 week | | | |
|  |  |  |  |  |  |  |  |  | 1-2 weeks | | | |
|  |  |  |  |  |  |  |  |  | >2 weeks | | | |
| **INFORMATION SYSTEMS, MEDICAL RECORDS AND REGISTRY**  **Super speciality/Medical College/Private multi-speciality hospital** | | | | | | | | | | | | |
| 1 | Does this health facility have the following health information systems  (Select: YES/NO) | | | Hospital Based Cancer Registry | | | | | | | | |
|  |  |  |  | Electronic Health  Records | | | | | | | | |
| 2 | Does this health facility have the following IT support (Select: YES/NO) | | | Clinical decision support | | | | | | | | |
|  |  |  |  | Electronic prescribing system | | | | | | | | |
|  |  |  |  | Computerized physician order | | | | | | | | |
|  |  |  |  | Telemedicine | | | | Consultation | | | | |
|  |  |  |  |  |  |  |  | Tele radiology | | | | |
|  |  |  |  |  |  |  |  | Tele-pathology | | | | |

**The proforma outlined here describes the data variables that were common/specific for a particular health facility.*
