# Supplementary material for: Situational Analysis of Cancer Care Services in India: Protocol for a Nationwide Cross-Sectional Health Systems Study
Source: JMIR Res Protoc. 2026 Oct 21;15:e99765. doi: 10.2196/99765 (PMC13686612; doi:10.2196/99765)
Supplement: Supplementary file 2 [file resprot-v15-e99765-s002.docx]

# STROBE Checklist

| **Section/Item** | **Recommendation** | **Reported (Yes/Partial/No)** | **Page/Section in Manuscript** | **Comments** |
| --- | --- | --- | --- | --- |
| Title/Abstract (1) | Indicate study design in title/abstract | Yes | Title, Abstract | Cross-sectional study identified in abstract. |
| Background (2) | Explain rationale | Yes | Introduction | Adequate rationale. |
| Objectives (3) | State objectives | Yes | Abstract, Introduction | Clearly stated. |
| Study design (4) | Present key elements | Yes | Methods | Cross-sectional descriptive. |
| Setting (5) | Describe setting | Yes | Methods | States/UTs and facilities. |
| Participants (6) | Eligibility and selection | Yes | Methods | Sampling framework described. |
| Variables (7) | Define outcomes/exposures | Yes | Methods | Indicators described. |
| Data sources (8) | Measurement methods | Yes | Methods | Electronic proformas. |
| Bias (9) | Address bias | Partial | Limitations | Reporting bias acknowledged. |
| Study size (10) | Explain study size | Yes | Methods | Sampling explained. |
| Quantitative variables (11) | Handling described | Partial | Methods | Mostly categorical. |
| Statistical methods (12) | Describe analyses | Yes | Methods | Descriptive + Chi-square. |
| Participants (13) | Numbers at each stage | Yes | Results | Coverage reported. |
| Descriptive data (14) | Participant characteristics | Yes | Results | Facility characteristics. |
| Outcome data (15) | Report outcomes | Yes | Results | Availability reported. |
| Main results (16) | Estimates and precision | Partial | Results | No effect estimates/CI. |
| Other analyses (17) | Subgroups/sensitivity | Partial | Results | Regional analyses only. |
| Key results (18) | Summarise findings | Yes | Discussion | Included. |
| Limitations (19) | Discuss limitations | Yes | Discussion | Comprehensive. |
| Interpretation (20) | Interpret results | Yes | Discussion | Balanced. |
| Generalisability (21) | Discuss external validity | Yes | Discussion | Nationwide applicability. |
| Funding (22) | Funding source | No | Not reported | Funding statement should be added. |
